# Spin‐Orbit Torque in Van der Waals‐Layered Materials and Heterostructures

**DOI:** 10.1002/advs.202100847

**Published:** 2021-07-29

**Authors:** Wei Tang, Haoliang Liu, Zhe Li, Anlian Pan, Yu‐Jia Zeng

**Affiliations:** ^1^ Key laboratory of Optoelectronic Devices and Systems of Ministry of Education and Guangdong Province College of Physics and Optoelectronic Engineering Shenzhen University Shenzhen 518060 China; ^2^ State Key Laboratory on Tunable Laser Technology Ministry of Industry and Information Technology Key Lab of Micro‐Nano Optoelectronic Information System School of Science Harbin Institute of Technology Shenzhen 518055 China; ^3^ Key Laboratory for Micro‐Nano Physics and Technology of Hunan Province College of Materials Science and Engineering Hunan University Changsha 410082 China

**Keywords:** magnetization switching, spin‐orbit coupling, spin‐orbit torques, spintronics, van der Waals‐layered materials

## Abstract

Spin‐orbit torque (SOT) opens an efficient and versatile avenue for the electrical manipulation of magnetization in spintronic devices. The enhancement of SOT efficiency and reduction of power consumption are key points for the implementation of high‐performance SOT devices, which strongly rely on the spin‐orbit coupling (SOC) strength and magnetic properties of ferromagnetic/non‐magnetic heterostructures. Recently, van der Waals‐layered materials have shown appealing properties for use in efficient SOT applications. On the one hand, transition‐metal dichalcogenides, topological insulators, and graphene‐based heterostructures possess appreciable SOC strength. This feature can efficiently converse the charge current into spin current and result in large SOT. On the other hand, the newly discovered layered magnetic materials provide ultra‐thin and gate‐tunable ferromagnetic candidates for high‐performance SOT devices. In this review, the latest advancements of SOT research in various layered materials are summarized. First, a brief introduction of SOT is given. Second, SOT studies of various layered materials and heterostructures are summarized. Subsequently, progresses on SOT‐induced magnetization switching are presented. Finally, current challenges and prospects for future development are suggested.

## Graphical abstract

Van der Waals‐layered materials and heterostructures show appealing advantages in improving spin‐orbit torque (SOT) efficiency. The recent SOT researches in various 2D materials are outlined. The current challenges and the future directions are also discussed. Van der Waals‐layered materials and heterostructures are expected to provide unprecedented opportunities in the fields of spintronics.

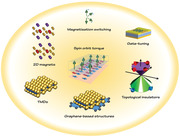

## 1 Introduction

The discoveries of giant magnetoresistance and tunnel magnetoresistance (TMR) have significantly improved the storage density of hard disk drives in the past decades.^[^ ^1^, ^2^, ^3^, ^4^, ^5^ ^]^ Meanwhile, with the rapid development of spintronics, novel spin‐based devices such as racetrack memory and magnetic random access memory (MRAM) have shown huge potentials in the post‐Moore era.^[^ ^6^, ^7^ ^]^ The power dissipation and operation speed of these spin‐based devices hinge on effective methods of controlling their magnetic states. However, traditional methods of manipulating magnetic states by a magnetic field is unpractical for the realization of high‐performance devices. To address this challenge, electrical current‐induced torques have been exploited to manipulate magnetization over the last decades. A remarkable progress is the application of the spin‐transfer torque (STT) effect, which is a phenomenon in which electric current is spin‐polarized by a ferromagnetic layer and the spin‐polarized electrons affect the magnetization of another ferromagnetic layer.^[^ ^8^, ^9^ ^]^ The utilization of STT conveniently facilitates the control of magnetization by electric current, and it has been used to fabricate commercial STT‐MRAMs. However, despite the promising merits of STT, the coupling of the read and write paths has a detrimental impact on device stability and performance.

Meanwhile, another spin‐torque mechanism, current‐induced spin‐orbit torque (SOT), has recently emerged as a promising technique for efficient magnetization manipulation.^[^ ^10^, ^11^, ^12^ ^]^ A typical SOT structure comprises a ferromagnetic (FM) layer and a normal metal (NM) layer. As charge current flows in the NM/FM heterostructure, the shunted part of the NM layer is conversed to spin‐polarized current owing to the bulk or interfacial spin‐orbit interaction. The spin current is then accumulated at the NM/FM interface and diffused into the adjacent FM layer. As a result, the spin‐polarized electrons exert a torque on the local magnetic moment by transferring their angular momentum. The fundamental origin of SOT is the strong spin‐orbit coupling (SOC) of the NM layer. SOT has been employed for the electrical manipulation of magnetization, such as, fast magnetization switching, high‐speed domain wall motion, and spin‐wave excitation.^[^ ^10^, ^11^, ^12^, ^13^, ^14^, ^15^, ^16^, ^17^, ^18^, ^19^ ^]^ Compared with STT devices, SOT devices have a number of advantages, including higher stability, faster response, and lower energy consumption.

For the efficient generation of spin current and magnetization switching, it is essential to enhance the SOC strength of the NM layer. A large number of FM and NM materials have been explored to construct FM/NM heterostructures. 5d heavy metals (HM), such as Pt, Ta, and W, were commonly used as spin sources in past studies because of their strong SOC.^[^ ^11^, ^12^, ^20^ ^]^ However, their SOT efficiencies, characterized by the spin Hall angle and the Rashba parameter, are usually limited (less than 1).^[^ ^21^, ^22^, ^23^ ^]^ Thus, the SOT efficiency is enhanced by engineering the heterostructure or using novel materials with strong SOC. As an alternative, 2D van der Waals (vdW)‐layered materials have recently been proposed as building blocks for SOT devices. Among the diverse vdW materials, transition metal dichalcogenides (TMDs) are promising candidates owing to their non‐trivial energy band structure, tunable conductivity, and strong SOC.^[^ ^24^, ^25^, ^26^ ^]^ TMDs exhibit several advantages in SOT applications. For instance, the SOT can be tuned via designing a crystalline symmetry and via the electric field.^[^ ^27^, ^28^ ^]^ Moreover, the SOT efficiencies of TMDs are larger than those of HMs, and such high SOT efficiencies can be scaled to the atomic layer limit. Additionally, semimetals such as WTe_2_ and PtTe_2_ have topological phases and strong SOCs with high electrical conductivities.^[^ ^26^, ^29^ ^]^

Another promising candidate is the family of topological insulators (TIs). Owing to their topologically non‐trivial surface states, TIs possess extremely large SOT efficiencies, which are several orders of magnitude larger than those of conventional HMs.^[^ ^30^, ^31^ ^]^ This large SOT efficiency allows for low‐power magnetization manipulation.^[^ ^32^ ^]^ In addition to TMDs and TIs, graphene‐based hybrid heterostructures have recently been proposed as suitable spin sources for SOT applications.^[^ ^33^, ^34^, ^35^ ^]^ Pristine graphene has been extensively studied as a spin transport channel owing to its long spin diffuse length and high electron mobility.^[^ ^36^ ^]^ Despite its considerably weak SOC, several studies have revealed that the enhancement of SOC in graphene is feasible via the extrinsic effect.^[^ ^37^, ^38^ ^]^ Moreover, the combination of graphene with other materials having strong SOC can give rise to graphene‐based heterostructures with strong SOC.^[^ ^38^, ^39^, ^40^ ^]^ Such enhanced SOC result in appreciable charge‐spin interconversions, and they can be further tuned by an electric field.^[^ ^35^, ^41^, ^42^ ^]^ Most importantly, in graphene‐based heterostructures, high electron mobility can be maintained while enhancing the SOC.

Very recently, atomically thin vdW magnetic crystals have been used to further enrich the family of vdW materials,^[^ ^43^, ^44^, ^45^, ^46^, ^47^, ^48^, ^49^, ^50^ ^]^ providing a perfect paradigm for the investigation of magnetism in the 2D limit. These vdW magnetic crystals offer unprecedented opportunities for SOT devices. Particularly, ferromagnetic vdW materials such as Cr_2_Ge_2_Te_6_, CrI_3_, and Fe_3_GeTe_2_ possess intrinsic perpendicular magnetic anisotropy (PMA).^[^ ^43^, ^45^, ^51^ ^]^ Their robust PMA can be preserved down to the monolayer limit, which makes their magnetic properties very sensitive to external stimuli. These features make them appealing for ultra‐thin magnetic layers in SOT devices.

As research interests in SOT devices based on vdW materials have increased, several excellent reviews have summarized recent work of SOT in vdW materials,^[^ ^47^, ^52^, ^53^, ^54^, ^55^, ^56^, ^57^, ^58^ ^]^ in which nonmagnetic vdW materials, such as, TMDs, TIs, and graphene, are mainly used as the spin current source or spin channel. In addition, magnetic vdW materials appear as promising candidates in SOT devices. Herein, we aim to provide an overview of state‐of‐the‐art progress of SOT in vdW materials and their heterostructures. First, we briefly discuss the generation of SOT. Second, we summarize SOT and charge–spin interconversion studies in non‐magnetic vdW materials and heterostructures. Third, we discuss magnetic vdW materials and their gate‐tunable magnetic properties, focusing on their SOT application. Afterward, we present SOT switching in various vdW heterostructures with low critical current density. Finally, we discuss current challenges and future perspectives for this emerging field.

## 2 Generation of Spin‐Orbit Torque

**Figure 1:**
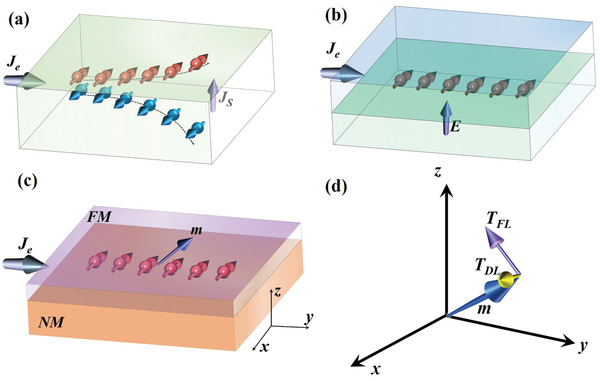
Illustration of SOC phenomena and SOT. a) Schematic of spin Hall effect. b) Schematic of Rashba–Edelstein effect. c) Schematic of spin accumulation at the FM/NM interface of a FM/NM heterostructure. d) Schematic of SOT. The purple and yellow arrows represent the field‐like and damping‐like torques, respectively.

Despite the controversy of the microscopic origin of SOT, it is generally agreed that SOT arises from spin accumulation at the NM/FM interface. Generally, spin accumulation comes from two different SOC phenomena, namely spin Hall effect (SHE) and Rashba–Edelstein effect (REE).^[^ ^59^, ^60^ ^]^ SHE is a bulk SOC effect, whereas REE is an interfacial SOC effect. As shown in **Figure** **1**a, SHE describes the behavior in which a charge current flowing in an NM layer generates a spin current owing to asymmetric spin deflection induced by SOC. In this case, the polarization direction is perpendicular to both the directions of the charge and spin currents. This generated spin current density is described as ***J***_s_∝(*ћ*/2*e*)*θ*_SH_ ***J***_e_ × ***σ***, where ***J***_s_ is the spin current density, ***J***_e_ is the charge current density, ***σ*** is the spin polarization direction, *ћ* is the reduced Planck's constant, *e* is the charge of an electron, and *θ*_SH_ is the spin Hall angle.

Compared to SHE, REE usually occurs at the interface or surface of a structure without spatial inversion symmetry. In this case, an internal electrical field ***E*** with a direction perpendicular to the film plane is generated at the interface/surface because of the spatial inversion symmetry breaking (Figure 1b). When an in‐plane charge current flows through the FM/NM heterostructure, the conduction electrons near the interface move in the electrical field ***E***, and they experience an effective magnetic field with a direction perpendicular to the current direction. This interfacial SOC (Rashba SOC)‐induced effective magnetic field is called Rashba field ***H***_R_. As a result, spin accumulation is induced in the direction perpendicular to both the directions of the charge current ***J*_e_** and out‐of‐plane electrical field ***E***(***E** = E*_z_ ***z***). This spin current density is described as ***J***_s_∝(*α*_R_/*ћe*)***z*** × ***J***_e_, where *α*_R_ is the Rashba parameter and ***z*** is the out‐of‐plane unit vector.

Typically, in an NM/FM heterostructure, when the charge current flows along the in‐plane of the film, the spin accumulation is in‐plane‐polarized. In the case of current along the *y*‐axis, the spin current is polarized along the x‐axis (Figure 1c). As the spin current diffuses into the adjacent FM layer, a SOT ***T***_SOT_ is exerted on the magnetization ***m***. This torque comprises two components, as shown in Figure 1d. The most general form is ***T***_SOT_ = ***T***_DL_ + ***T***_FL_, where ***T***_DL_ = *τ*_DL_ ***m*** × (***m*** × ***ξ***) is the longitudinal (Slonczewski‐like) torque (also called damping‐like SOT), ***T***_FL_ = *τ*_FL_ ***m*** × ***ξ*** is the transverse torque (also called field‐like SOT), ***ξ*** is the polarization vector, *τ*_DL_ and *τ*_FL_ are the magnitudes of the damping‐like and field‐like torques, respectively. Both SHE and REE can contribute *τ*_DL_ and *τ*_FL_. Distinguishing the contribution of SHE and REE to *τ*_DL_ and *τ*_FL_ is important to understand the underneath mechanism of SOT. The NM thickness dependence of SOT is a powerful tool to clarify the contribution from SHE and REE. Because in the simple model the interfacial Rashba effect is expected to be independent on the NM thickness, while the bulk SHE should scale with the NM thickness. At present, the exact mechanism of bulk and interfacial effects to *τ*_DL_ and *τ*_FL_ still requires a better understanding. However, it can be agreed that the *τ*_DL_ in most systems is strongly associated with SHE, while the important contribution of REE to the origin of the *τ*_FL_ is also well understood.

To obtain large spin current density and strong SOT, materials with large spin Hall angles and Rashba parameters, such as Pt and Ta, are preferred for FM/NM heterostructures. In addition to SHE and REE, topologically non‐trivial surface state has recently been found to significantly contribute to the spin current. Moreover, unconventional SOT has also been observed in some TMDs. For comparison, spin conductivity *σ*_sh_ is used here. The relationship between the spin Hall angle and spin conductivity is *θ*_SH_ = (2*e*/*ћ*) *σ*_sh_/*σ*_e_, where *σ*_e_ is electrical conductivity.

Spin Hall angle and Rashba parameter characterize the charge‐spin conversion efficiency, and they strongly depend on the SOC strengths in the NM layer and NM/FM interface. They are sometimes also referred to as charge–spin conversion efficiency or SOT efficiency *χ*_SOT_. It should be noted that the SOT efficiency is different from the spin Hall angle and the charge‐spin conversion efficiency. In SHE induced SOT picture, if the spin‐memory loss is negligible or the interface is transparent, the SOT efficiency is equal to the spin Hall angle. The relationship of the SOT efficiency and the spin Hall angle can be expressed as *χ*_SOT_ *= T*_int_ *θ*_SH_, where *T*_int_ is the interface spin transparency.^[^ ^61^ ^]^ In this review, for simplicity, we will use SOT efficiency to refer to the spin Hall angle and charge–spin conversion efficiency, unless otherwise specified.

## 3 Non‐Magnetic Van der Waals Materials and Heterostructures for Spin‐Orbit Torque

### 3.1 Transition Metal Dichalcogenides

Monolayer TMDs, such as group‐VI dichalcogenides MX_2_ (M = Mo, W; X = S, Se), are regarded as intrinsically strong SOC materials that can serve as highly efficient spin sources.^[^ ^62^, ^63^, ^64^, ^65^ ^]^ Their high SOC strength has two important origins. First, the heavy metal element, such as Mo and W, can generate a strong SOC.^[^ ^66^ ^]^ Second, Rashba‐type or Zeeman‐type spin splitting can be induced at their surface owing to the inversion symmetry breaking, which gives rise to a strong SOC.^[^ ^24^, ^60^ ^]^ These two contributions enable TMDs to have appreciable SOT efficiencies that induce high SOTs in TMDs/ferromagnetic structures.

**Figure 2:**
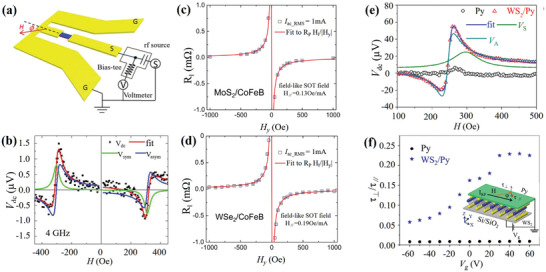
a) Schematic of ST‐FMR experimental setup for MoS_2_/Py heterostructure. b) Typical field scan of ST‐FMR signals. The green and blue lines are fitted by symmetric and antisymmetric Lorentzian functions, respectively. The red line is fitted by the sum of the symmetric and antisymmetric Lorentzian functions. a,b) Reproduced under the terms of the Creative Commons CC‐BY license.^[^ ^68^ ^]^ Copyright 2016, The Authors. Published by AIP Publishing. Extracted R_∥_ as a function of the external magnetic field along the ±y direction for the bilayer c) MoS_2_/CoFeB and d) WSe_2_/CoFeB. The red solid curve is the fitting curve. c,d) Reproduced with permission.^[^ ^71^ ^]^ Copyright 2016, American Chemical Society. e) Comparison of the ST‐FMR resonance signals *V*_dc_ of bilayer WS_2_/Py and pure Py at a fixed frequency of 5 GHz and fixed power of 10 dBm. f) Torque ratio *τ*_∥_/*τ*_⊥_ dependence of *V*_g_ for Py and bilayer WS_2_/Py. Inset: Schematic of the bilayer WS_2_/Py device geometry. *V*_g_ is applied through the SiO_2_ dielectric layer. e,f) Reproduced with permission.^[^ ^28^ ^]^ Copyright 2018, American Chemical Society.

Experimentally, a spin pumping measurement has shown that the spin‐charge conversion efficiency of MoS_2_ is as large as 12.7.^[^ ^67^ ^]^ Subsequently, current‐induced SOT was reported in a monolayer MoS_2_/permalloy (Py) heterostructure.^[^ ^68^ ^]^ Meanwhile, different SOT components can be identified and distinguished using spin‐torque‐ferromagnetic resonance (ST‐FMR) technique (**Figure** **2**a). In this technique, magnetization precession is excited by the current‐induced SOT and Oersted‐field torque *τ*_h_. Magnetization precession results in an alternating magnetoresistance. The coupling of alternating magnetoresistance and microwave current generates a mixed DC voltage comprising symmetric and antisymmetric Lorentzian functions. Both *τ*_FL_ and *τ*_h_ contribute to the antisymmetric Lorentzian peak, whereas *τ*_DL_ contributes to the symmetric Lorentzian peak. More details of the ST‐FMR and other measurement techniques can be found in another study.^[^ ^69^, ^70^ ^]^ As shown in Figure 2b, the ST‐FMR signals of the MoS_2_/Py sample exhibited a relatively large symmetric peak. The calculated amplitude of the symmetric peak was approximately four times that of the antisymmetric peak. These results suggest that *τ*_DL_ dominates in the MoS_2_/Py heterostructure. The authors attribute the *τ*_DL_ to the interfacial effect‐induced SOC. However, Shao et al. conducted a similar SOT study in a monolayer (MoS_2_ or WSe_2_)/CoFeB heterostructure and observed different results.^[^ ^71^ ^]^ They quantitatively measured the current‐induced SOT using second‐harmonic Hall (SHH) approach, and found that both the MoS_2_/CoFeB and WSe_2_/CoFeB samples exhibited a large *τ*_FL_, whereas *τ*_DL_ was negligible, as shown in Figure 2c,d. The origin of the large *τ*_FL_ can be explained by REE, as *τ*_FL_ is much larger than *τ*_DL_ in this case. Furthermore, the extracted spin conductivities of MoS_2_ and WSe_2_ were 2.9 × 10^3^ and 5.5 × 10^3^ (ℏ/2*e*)(Ωm)^−1^, respectively, which suggest that monolayer MoS_2_ and WSe_2_ have appreciable SOT efficiencies. For the discrepancy between MoS_2_/CoFeB and MoS_2_/Py, the observed large symmetric peak possibly derives from the inverse REE induced by spin pumping rather than the SOT induced by the *rf* spin current. Another possible origin is that the ferromagnetic materials play a key role in the current‐induced SOT. More detailed experimental work is required to identify different contributions.

In addition to MoS_2_ and WSe_2_,^[^ ^72^ ^]^ current‐induced SOT has also been investigated in a monolayer WS_2_/Py heterostructure using ST‐FMR, and the ST‐FMR spectra of the WS_2_/Py heterostructure exhibited a large symmetric peak (Figure 2e).^[^ ^28^ ^]^ The authors attribute this symmetric peak to the current‐induced *τ*_DL_. Interestingly, the ratio of the symmetric peak to the antisymmetric peak can be tuned up to a factor of four within a gate voltage range of −60 to 60 V, which suggests the tunability of different SOT components (Figure 2f). This SOT modulation via the gate voltage is explained by the fact that the carrier density, as well as, the interface transparency and depletion layer width can be controlled by the electric field.

TMDs such as MoS_2_ have moderate SOT efficiencies and high resistivities. Moreover, the spin current generated by the in‐plane current is commonly in‐plane‐polarized. Therefore, the induced *τ*_DL_ is oriented in the sample plane. As a sequence, one must apply an in‐plane bias field to break the symmetry along the current direction and determine the SOT switching of the perpendicular magnetization.^[^ ^10^, ^11^, ^12^, ^20^ ^]^ Meanwhile, an out‐of‐plane‐polarized spin current will produce an out‐of‐plane antidamping SOT *τ*_B_. Different from the conventional in‐plane SOT *τ*_DL_, this unconventional SOT *τ*_B_ is more energy‐efficient for PMA magnet switching.^[^ ^27^ ^]^ By utilizing *τ*_B_, the magnetization can be switched without applying an in‐plane bias field. For common materials with high symmetries, such as Pt and MoS_2_, out‐of‐plane‐polarized spin current cannot be generated by an in‐plane charge current. However, out‐of‐plane‐polarized spin current is allowed in TMDs with reduced crystalline symmetries.

**Figure 3:**
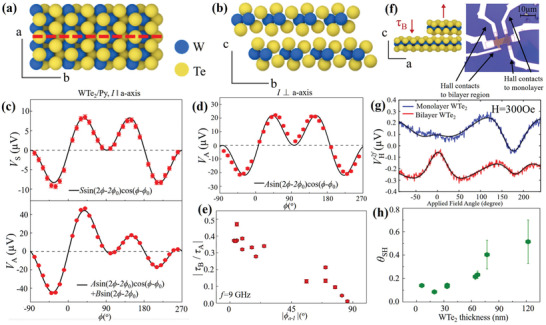
a,b) Crystal structure in a WTe_2_ surface. The surface structure has mirror symmetry with respect to the bc‐plane (dashed line). Different ST‐FMR peak components versus in‐plane magnetic field angle *φ*. c) Symmetric (top) and antisymmetric (bottom) components of WTe_2_ (5.5 nm)/Py (6 nm) heterostructure with current along the a‐axis. d) Antisymmetric component of a WTe_2_ (15 nm)/Py (6 nm) heterostructure with current along the b‐axis. e) Ratio of the torque *τ*_B_ to the out‐of‐plane effective field torque *τ*_A_ versus the angle between the a‐axis and the applied current. a–e) Reproduced with permission.^[^ ^27^ ^]^ Copyright 2016, Springer Nature. f) Optical picture of a device with a monolayer step and schematic of the WTe_2_ crystal structure, showing that the surface structure is rotated by 180° across a monolayer step. g) Second‐harmonic Hall data for a WTe_2_/Py device for a region of the sample with a monolayer‐thick WTe_2_ layer (top curve, blue) and for a different region of the same sample with bilayer WTe_2_ (bottom curve, red), as a function of the angle of the applied magnetic field (defined relative to the current flow direction). f,g) Reproduced with permission.^[^ ^75^ ^]^ Copyright 2017, American Physical Society. h) Thickness‐dependent SOT efficiency of WTe_2_. Reproduced with permission.^[^ ^29^ ^]^ Copyright 2019, Springer Nature.

Semimetal WTe_2_ is another attractive spin‐source material owing to its strong SOC and relatively high electron mobility.^[^ ^73^, ^74^ ^]^ Moreover, compared to MoS_2_, 1T'‐WTe_2_ has lower crystalline symmetry. As illustrated in **Figure** **3**(a) and (b), WTe_2_ has only one mirror plane perpendicular to the b‐axis, while the screw symmetry along the c‐axis and a mirror symmetry with respect to the ac‐plane are broken. This lateral symmetry breaking may give rise to out‐of‐plane *τ*_B_ in the WTe_2_/FM heterostructure. Experimentally, MacNeill et al. observed a large out‐of‐plane *τ*_B_ in a bilayer WTe_2_/Py.^[^ ^27^ ^]^ Using ST‐FMR technique, conventional SOT and out‐of‐plane *τ*_B_ were identified from the field dependence of SOT signals. When *rf* current is along the a‐axis (low‐symmetry axis), an additional angular dependence term sin (2*φ*) suggests the existence of *τ*_B_ apart from the contribution of the conventional SOT (Figure 3c). Once the applied *rf* current is parallel to the b‐axis (high‐symmetry axis), this additional angular dependence vanishes and only conventional SOT is present (Figure 3d). The full angle dependence also shows a reduction in *τ*_B_ as the current is tuned from the a‐axis to the b‐axis (Figure 3e). MacNeill et al. explained that these results were due to the lateral symmetry breaking at the interface, which was further confirmed by their subsequent work.^[^ ^75^ ^]^ In a WTe_2_/Py device with a monolayer step (Figure 3f), the angle dependence of the SHH voltage indicates the inversed sign of *τ*_B_ for monolayer and bilayer WTe_2_ (Figure 3g), which is consistent with non‐symmorphic symmetries in the WTe_2_. As shown in Figure 3f, the adjacent WTe_2_ samples are related by a 180° rotation around the c‐axis, and *τ*_B_ changes sign because of its non‐twofold symmetry. Owing to the strong SOC, the spin conductivities of the conventional damping‐like SOT and field‐like SOT are estimated as 8 ± 2 × 10^3^ and 9 ± 3 × 10^3^ (ℏ/2*e*)(Ωm)^−1^, respectively. Moreover, the spin conductivity of the unconventional torque *τ*_B_ is also appreciable, with a value of 3.6 ± 0.8 × 10^3^ (ℏ/2*e*)(Ωm)^−1^. For these three SOT components, their independency on thickness indicates the interface origin. It is worth noting that the bulk SHE also plays a key role in a much thicker WTe_2_. Shi et al. recently reported the dependence of SOT on thickness in a thick WTe_2_/Py heterostructure using ST‐FMR and magnetization switching.^[^ ^29^ ^]^ Both measurements showed that the SOT efficiency increased as the WTe_2_ thickness increased (Figure 3h), suggesting a bulk‐like contribution to the SOT. Additionally, the SOT strength was significantly enhanced and the spin conductivity was approximately 6 × 10^4^ (ℏ/2*e*)(Ωm)^−1^.

**Figure 4:**
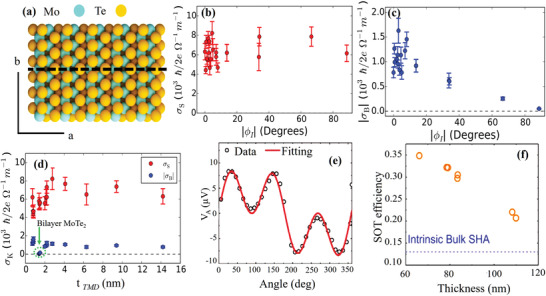
a) Structure of a monoclinic MoTe_2_ crystal (*β* phase) in the a,b‐plane, where the a‐c mirror plane is depicted by the black dashed line. Spin conductivities versus the angle |*φ*_I_| between the b‐axis and applied current for b) *τ*_DL_ and c) *τ*_B_. d) *τ*_B_ (*σ*_B_) and *τ*_DL_ (*σ*_S_) spin conductivities as functions of MoTe_2_ thickness with current along the b‐axis. a–d) Reproduced with permission.^[^ ^66^ ^]^ Copyright 2019, American Physical Society. e) Antisymmetric component versus the in‐plane magnetic field angle with current applied in parallel to the a‐axis. f) Thickness dependence of the experimental SOT efficiency and the intrinsic bulk spin Hall angle of MoTe_2_ slabs calculated at the Fermi level. e,f) Reproduced with permission.^[^ ^77^ ^]^ Copyright 2020, Wiley‐VCH.

Furthermore, isostructural *β*‐MoTe_2_ is another TMD with strong SOC and low symmetry. Similar to WTe_2_, in the surface symmetry of monolayer *β*‐MoTe_2_, only one mirror plane is perpendicular to the b‐axis (**Figure** **4**a). However, an important distinction is that MoTe_2_ has bulk inversion symmetry. Similarly, conventional SOT and unconventional torque *τ*_B_ can be observed in a bilayer MoTe_2_/Py using ST‐FMR method.^[^ ^76^ ^]^ The unconventional torque *τ*_B_ is also generated only when the current is along the a‐axis. This work demonstrates that the bulk symmetry breaking is not a necessary requirement for the generation of *τ*_B_. The spin conductivity of the conventional SOT in MoTe_2_ is comparable with that in WTe_2_ (Figure 4b), while the *τ*_B_ spin conductivity in MoTe_2_ is relatively weak, which is about three times smaller than that in WTe_2_ (Figure 4c). However, the significant reduction in *τ*_B_ in bilayer MoTe_2_ devices is yet to be understood (Figure 4d). Meanwhile, Shi et al. recently showed that ***τ***_B_ is negligible in a thicker MoTe_2_ flake, as shown in Figure 4e.^[^ ^77^ ^]^ The SOT efficiency became smaller as the thickness of the MoTe_2_ films increased, which is in contrast to that of WTe_2_ (Figure 4f). The maximum value of the SOT efficiency in MoTe_2_ is similar to the result obtained for hybrid MoTe_2_‐graphene lateral spin valves.^[^ ^78^ ^]^ The authors speculate that both interfacial REE and bulk SHE contributions play key roles in the generation of SOT. Current‐induced SOT has also been investigated in TaTe_2_ with low‐crystal symmetry.^[^ ^79^ ^]^ However, neither unconventional nor conventional SOT was detected within the experimental resolution.

Spin‐current source materials are expected to simultaneously possess high conductivity and strong SOC. Unfortunately, TMDs usually have small conductivities, such as semiconductor MoS_2_ with *σ*_e_ ≈ 10^−3^–10^−6^ (Ωm)^−1^,^[^ ^34^, ^80^ ^]^ and semimetal WTe_2_ with *σ*_e_ ≈ 10^5^ (Ωm)^−1^,^[^ ^73^, ^74^ ^]^ resulting in a small effective charge current. Recently, several TMDs, such as, NbSe_2_, TaS_2_, and PtTe_2_, have been found to be ideal spin‐source materials owing to their high conductivity and strong SOC.

**Figure 5:**
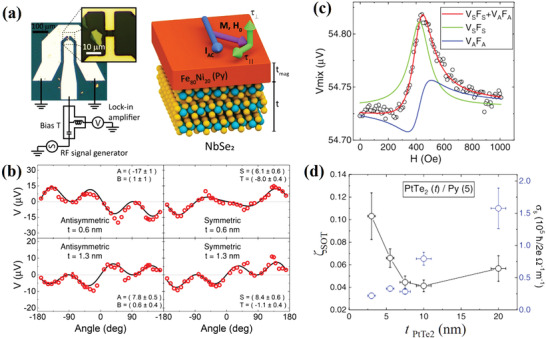
a) Schematics of a typical NbSe_2_ device with the measuring circuit and a NbSe_2_/Py heterostructure. b) Antisymmetric and symmetric components of the ST‐FMR resonance versus the magnetic field angle for devices with one and two NbSe_2_ monolayers, respectively. a,b) Reproduced with permission.^[^ ^82^ ^]^ Copyright 2018, American Chemical Society. c) Typical ST‐FMR signal and the corresponding Lorentzian fitting for a PtTe_2_ (5 nm)/Py (2.5 nm) device. d) Thickness dependence of SOT efficiency *ξ*_SOT_ and spin conductivity *σ*_s_ for damping‐like torque *τ*_DL_. c,d) Reproduced with permission.^[^ ^86^ ^]^ Copyright 2020, Wiley‐VCH.

NbSe_2_ is a fully metallic TMD with strong SOC and *σ*_e_ ≈ 6 × 10^5^ (Ωm)^−1^.^[^ ^81^ ^]^ Using the ST‐FMR approach, conventional SOT was observed in a NbSe_2_/Py heterostructure (**Figure** **5**a), and the corresponding spin conductivity was estimated as ≈4 × 10^4^ (ℏ/2*e*)(Ωm)^−1^.^[^ ^82^ ^]^ Interestingly, based on the symmetry requirements, unconventional SOT was prohibited in the heterostructure because of its highly symmetric structure. However, an unconventional in‐plane field‐like SOT with the form ***m*** × ***z*** was identified from the angular dependence (Figure 5b). This unexpected SOT is attributed to the strain effect, which may be present during the device fabrication, thereby breaking the symmetry and allowing the unconventional in‐plane field‐like SOT. Similarly, 1T‐TaS_2_ possesses a very large electric conductivity, *σ*_e_ ≈ 5.9 × 10^6^ (Ωm)^−1^. Furthermore, considerable SOT has been observed in TaS_2_/Py heterostructure using ST‐FMR and angle‐dependent planar Hall effect measurements,^[^ ^83^ ^]^ in which current‐induced *τ*_DL_ was found to dominate. This large *τ*_DL_ is attributed to the interplay between interfacial SOC and crystal symmetry. The spin conductivity of the TaS_2_/Py heterostructure was calculated as ≈14.9 × 10^5^ (ℏ/2*e*)(Ωm)^−1^, which is comparable to that of Pd_1−_ *_x_*Pt*_x_* alloy.^[^ ^84^ ^]^ Meanwhile, this estimated SOT efficiency value is larger than that of *β*‐Ta.^[^ ^11^ ^]^

Different from the metallic NbSe_2_ and TaS_2_, PtTe_2_ is a semimetal TMD that possesses room‐temperature high conductivity, *σ*_e_ ≈ 3.3 × 10^6^ (Ωm)^−1^.^[^ ^85^ ^]^ Xu et al. recently studied the SOT in a PtTe_2_/Py heterostructure and observed that the ST‐FMR signal had a relatively large symmetric peak (Figure 5c).^[^ ^86^ ^]^ The authors carefully excluded the contribution of spin pumping. Therefore, the symmetric contribution was attributed to the current‐induced *τ*_DL_. The spin conductivity of PtTe_2_ ranged from 0.22 × 10^5^ to 2 × 10^5^ (ℏ/2*e*)(Ωm)^−1^, which is comparable with those of Pt and Bi_2_Se_3_ at room temperature. Additionally, both the SOT efficiency and spin conductivity exhibited nonmonotonic thickness dependences, as shown in Figure 5d. These results suggest that there are several different mechanisms of SOT generation. Meanwhile, interfacial REE is ruled out because of the negligible *τ*_FL_. Thus, bulk SHE and topological surface state (TSS) may contribute to the observed SOT. However, an understanding of the exact mechanism needs further investigation.

Some of the aforementioned TMDs are also categorized as Weyl or Dirac semimetals.^[^ ^26^, ^87^, ^88^, ^89^, ^90^, ^91^, ^92^, ^93^, ^94^, ^95^ ^]^ Similar to TIs, these topological semimetals possess TSSs, in which strong SOC and large SOT are expected. Indeed, SOT originating from TSSs was recently revealed in a Weyl semimetal WTe_2_/Py heterostructure,^[^ ^29^, ^96^ ^]^ in which both *τ*_DL_ and *τ*_FL_ were observed. The spin conductivity of the *τ*_FL_ was significantly enhanced at low temperature when the applied current was along the b‐axis of WTe_2_.^[^ ^96^ ^]^ The enhanced spin conductivity is interpreted by the spin‐momentum locking in the topological Fermi arc surface state. Apart from WTe_2_, current‐induced SOT has also been studied in other Weyl and Dirac semimetal TMDs, such as MoTe_2_ and PtTe_2_. These Weyl and Dirac semimetals show potential and interesting applications in low‐power SOT devices.

### 3.2 Topological Insulators

**Figure 6:**
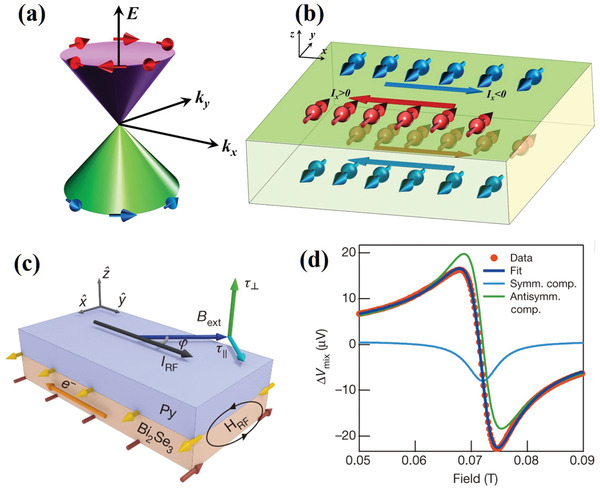
a) Schematic of the spin‐momentum‐locked spin texture of the TSS in TI. b) Schematic of surface spin density on two opposite surfaces for a charge current flowing along the −x direction (*I*_x_ < 0, blue arrow) and a charge current flowing along the +x direction (*I*_x_ > 0, red arrow). c) Schematic of a bilayer Bi_2_Se_3_/Py heterostructure and its coordinate system. The yellow and red arrows denote the spin moment directions. d) ST‐FMR signal at room temperature with 8‐GHz microwave frequency for a Bi_2_Se_3_ (8 nm)/Py (16 nm) sample. c,d) Reproduced with permission.^[^ ^30^ ^]^ Copyright 2014, Springer Nature.

TIs are a class of quantum materials that have insulating bulk states owing to the presence of bulk bandgaps. However, their topologically protected surfaces or edge states are metallic conducting. TI materials were initially reported in 2D systems. Later, the first 3D TI, Bi_0.9_Sb_0.1_, was confirmed by angle‐resolved photoemission spectroscopy.^[^ ^97^ ^]^ Since then, more 3D TIs with larger bulk bandgaps have been discovered, including Bi_2_Se_3_, Bi_2_Te_3_, and Sb_2_Te_3_.^[^ ^98^, ^99^, ^100^ ^]^ In these 3D TIs, the TSS results in strong coupling of the electron spin and its momentum. Therefore, the conductive electrons are highly spin‐polarized and the spin direction is locked to its momentum, which is referred to as spin‐momentum locking (**Figure** **6**a,b). Owing to this exotic property, the spin polarization efficiency of the TSS is extremely large, which makes TIs promising for SOT devices.

Motivated by these unique advantages, experimental studies began to focus on current‐induced SOT in various TI/FM heterostructures.^[^ ^101^, ^102^, ^103^, ^104^, ^105^, ^106^, ^107^, ^108^ ^]^ Mellnik et al. first studied current‐induced SOT in Bi_2_Se_3_/Py heterostructure by ST‐FMR (Figure 6c).^[^ ^30^ ^]^ As shown in Figure 6d, the ST‐FMR signal exhibited a large symmetric peak, which suggests a considerable conventional damping‐like SOT *τ*_DL_. It should be emphasized that TI has large resistivity, indicating that although its *τ*_DL_ spin conductivity (1–2 × 10^5^ (ℏ/2*e*)(Ωm)^−1^) is comparable to that of bilayer HM/FM heterostructures, the magnitude of *τ*_DL_ per unit current is much larger than that of heavy metals. Additionally, the out‐of‐plane *τ*_FL_ was significantly large with a spin conductivity of ≈1.5 × 10^5^ (ℏ/2*e*)(Ωm)^−1^ (note that the *τ*_FL_ in heavy metals is usually negligible because of its SHE origin). Such a large *τ*_FL_ cannot be explained by *τ*_h_. This SOT configuration agrees with the model of SOT derived from the TSS of TIs.

**Figure 7:**
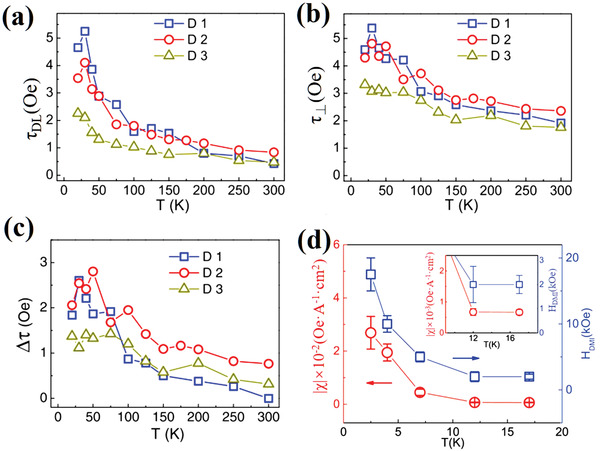
Temperature dependence of SOT in a Bi_2_Se_3_/CoFeB device. a) In‐plane torque *τ*_DL_. b) Total out‐of‐plane torque *τ*_⊥_. c) Out‐of‐plane torque Δ*τ*. a–c) Reproduced with permission.^[^ ^101^ ^]^ Copyright 2015, American Physical Society. d) Temperature dependence of saturated SOT efficiency (*χ*_sat_) and *H*_DMI_, where *H*_DMI_ is the Dzyaloshinskii–Moriya interaction effective field. Inset: Zoomed‐in figure of data points at 12 and 17 K. Reproduced with permission.^[^ ^109^ ^]^ Copyright 2020, Wiley‐VCH.

Subsequently, current‐induced SOT was further investigated in various TI/FM heterostructures. The roles of different channels were verified to obtain high SOT efficiency and to tune the SOT. Meanwhile, TSS, bulk state, 2D electron gas usually coexist in TI materials.^[^ ^102^, ^105^ ^]^ The two latter states could make inevitable contributions to the spin current while reducing the role of TSS at room temperature. More importantly, TSS is sensitive to temperature, which may result in weak SOT at higher temperatures. As shown in **Figure** **7**a–c, Wang et al. found that the TSS‐induced SOT nonlinearly decreased as the temperature increased.^[^ ^101^ ^]^ The *τ*_DL_ at low temperature was ≈10 times larger than that at room temperature (Figure 7a). Meanwhile, a significant out‐of‐plane torque Δ*τ* was observed when the temperature was below 50 K (Figure 7c). This strong temperature dependence of SOT in TSS was also recently demonstrated in TI/magnetic‐TI by transport and optical methods.^[^ ^109^ ^]^ The maximum SOT effective field was obtained at a low temperature of 2.5 K, and the value drastically decreased with increasing temperature (Figure 7d). Such temperature evolution is attributed to a higher spin generation efficiency in the TSS at a lower temperature. These results suggest that increasing TSS contribution is highly important for maximizing the SOT.

**Figure 8:**
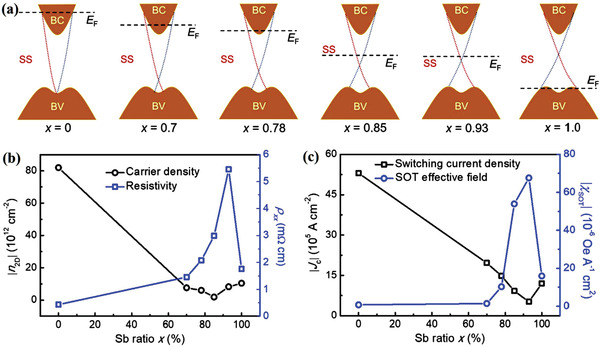
a) Schematic of the Fermi‐level positions for different Sb ratios (*x* = 0, 0.7, 0.78, 0.85, 0.93, 1.0) of (Bi_1−_ *_x_*Sb*x*)_2_Te_3_, which are estimated from the 2D carrier density |*n*_2D_| and resistivity *ρxx*. b) |*n*_2D_| and *ρxx* versus the Sb ratios in (Bi_1−_ *_x_*Sb*x*)_2_Te_3_. c) Switching current density |*J*_c_| and SOT‐induced effective field |*χ*_SOT_| as a function of the Sb ratios. Reproduced with permission.^[^ ^106^ ^]^ Copyright 2019, American Physical Society.

Increasing TSS contribution is also feasible by optimizing the TI thickness and controlling the Fermi‐level position.^[^ ^102^, ^110^ ^]^ A recent study revealed a clear relationship between SOT, the bulk state, and TSS by tuning the Fermi‐level position.^[^ ^95^ ^]^ As shown in **Figure** **8**a–c, the Fermi level changed from the bulk conduction band to the bulk valence band as the Sb content increased. When the Fermi level was close to the Dirac point, the current‐induced SOT was significantly enhanced (Figure 8c). This work clearly demonstrates that a dominating TSS plays a key role in the efficient generation of SOT.

**Figure 9:**
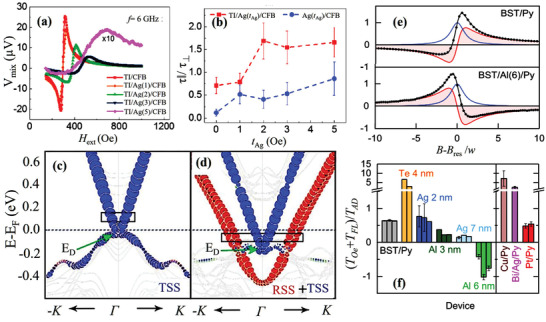
a) ST‐FMR signals for Bi_2_Se_3_/Ag(*t*_Ag_)/CoFeB at 6 GHz. The signal for the Ag (5 nm) sample is scaled by ten times. b) Ratio (*τ*_∥_/*τ*_⊥_) of in‐plane torque *τ*_∥_ to out‐of‐plane torque *τ*_⊥_ obtained for both Bi_2_Se_3_/Ag(*t*_Ag_)/CoFeB and Ag(*t*_Ag_)/CoFeB samples. Bi_2_Se_3_ (10 QL) and CoFeB (7 nm) (QL: quintuple layer) are denoted as Bi_2_Se_3_ and CFB, respectively. c,d) Band structures of Bi_2_Se_3_ (6 QL)/Ag (0 nm) and Bi_2_Se_3_ (6 QL)/Ag (0.95 nm). The weight of the first and second quintuple layers is highlighted by the size of the circles. The RSSs are in red and the TSSs are in blue. a–d) Reproduced with permission.^[^ ^107^ ^]^ Copyright 2018, American Physical Society. e) Normalized ST‐FMR signals with and without 6‐nm Al spacer layers inserted at the (Bi_1−_ *_x_*Sb*_x_*)_2_Te_3_/Py interface. The fitting curves (black) include the sum of the symmetric (blue) and antisymmetric (red) Lorentzians. f) (*T*_Oe_ + *T*_FL_)/*T*_AD_ for various devices and multilayers, where *T*_Oe_, *T*_FL_, and *T*_AD_ are the Oersted‐field torque, field‐like SOT, and damping‐like torque, respectively. Each bar corresponds to one device, and the error bars represent standard deviations in the range of 5–15 GHz. e,f) Reproduced with permission.^[^ ^108^ ^]^ Copyright 2020, American Chemical Society.

Notably, some works also showed that the bulk SOC and interfacial Rashba effect may play significant roles in the SOT in TI.^[^ ^107^, ^111^ ^]^ Particularly, the interface effect could be dominating in TI/FM heterostructures. The influence of interfacial Rashba effect was recently observed in a Bi_2_Se_3_/Ag/CoFeB heterostructure.^[^ ^107^ ^]^ As the Ag thickness increased, the dominating SOT changed from *τ*_FL_ to *τ*_DL_ (**Figure** **9**a,b), which is attributed to the large Rashba splitting bands. Different from the conventional Rashba effect, such Rashba splitting states are located outside the TSS and have the same net spin polarization direction as the TSS (Figure 9c,d). Furthermore, this interfacial effect of Bi_2_Se_3_/Ag can exceed the TSS, which plays a dominating role in the SOT efficiency. Moreover, the material intermixing at the interface of the TI/FM is also important, as it leads to significant variations in the nature of the SOT (Figure 9e,f). Therefore, different SOTs can be tuned via interface engineering.^[^ ^108^ ^]^

**Figure 10:**
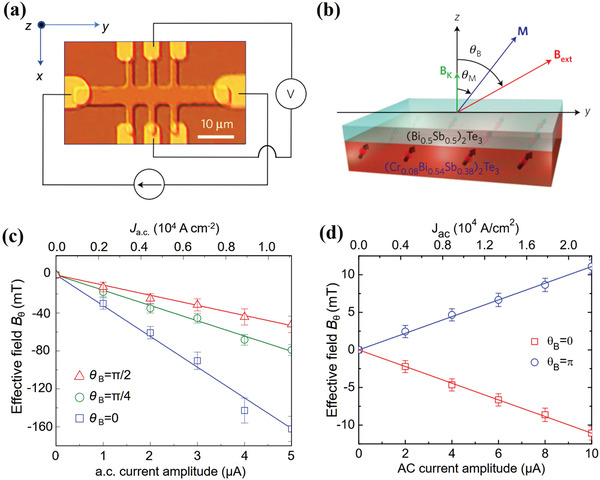
a) Picture of the Hall bar device with schematic illustrations of the Hall measurement set‐up. b) 3D schematic of the bilayer heterostructure. The top layer (light blue) shows the three quintuple layers (Bi_0.5_Sb_0.5_)_2_Te_3_ and the bottom layer (light red) shows the six quintuple layers (Cr_0.08_Bi_0.54_Sb_0.38_)_2_Te_3_. SOT effective fields as a function of the AC current amplitude *I*_ac_ for different magnetic field angles *θ*_B_. The straight lines are linear fittings. The error bars show the standard errors. In all the rotation experiments, the *B*_ext_ field magnitude is fixed at 2 T and the temperature is kept at 1.9 K. c) Damping‐like SOT effective field with magnetic field angle in the *yz*‐plane. d) Field‐like SOT effective field with magnetic field angle in the *xz*‐plane. Reproduced with permission.^[^ ^114^ ^]^ Copyright 2014, Springer Nature.

**Figure 11:**
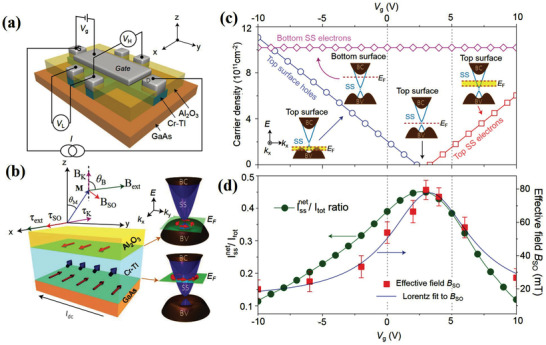
a) 3D schematic of the Hall bar structure made from the Al_2_O_3_ (20 nm)/(Cr_0.16_Bi_0.50_Sb_0.42_)_2_Te_3_ (7 nm)/GaAs (substrate) stack with a top Au gate electrode (light grey). A standard four‐point measurement set‐up is displayed. A gate voltage *V*_g_ can be applied between the top gate and the source contact. b) 3D schematic of the Al_2_O_3_/(Cr_0.16_Bi_0.50_Sb_0.42_)_2_Te_3_/GaAs structure. Right: Top and bottom surface band structures. BC is the bulk conduction band; BV is the bulk valence band; SS is the topological surface state; and *E*_F_ is the Fermi level. c,d) Top and bottom surface carrier densities as functions of *V*_g_. The insets show the top and bottom surface band structures in different gate voltage regions. The yellow shading in the top surface band structure shows the tuneable range of the top surface *E*_F_ within the corresponding gate voltage region. Lower panel: Comparison between the ratio *I*_net_ SOT effective field *B*_SO_ as a function of *V*_g_. Reproduced with permission.^[^ ^32^ ^]^ Copyright 2016, Springer Nature.

In the heterostructures of high‐resistive TI and low‐resistive metal magnets, most of the electrical current is shunted through the magnetic metal layer, which leads to a weak SOT strength. By replacing the metal magnet with a magnetic insulator, the SOT strength can be strongly enhanced. The recently demonstrated magnetic TI is a choice for the magnetic layer.^[^ ^112^, ^113^ ^]^ Indeed, a large SOT has been observed in TI/magnetic‐TI heterostructures using SHH method, in which (Bi_0.5_Sb_0.5_)_2_Te_3_ and Cr‐doped (Bi_0.5_Sb_0.5_)_2_Te_3_ served as the spin source and magnetic layer, respectively (**Figure** **10**a,b).^[^ ^114^ ^]^ Different from Bi_2_Se_3_‐based structures, *τ*_DL_ dominates in (Bi_0.5_Sb_0.5_)_2_Te_3_, which is 30 times larger than the strength of *τ*_FL_ (Figure 10c,d). The estimated SOT effective field and SOT efficiency are three orders of magnitude larger than that of heavy metals at low temperature. Such a large SOT is attributed to the spin‐momentum locking mechanism of TSS. Moreover, a large and tunable SOT has been shown in a Cr‐doped magnetic topological insulator.^[^ ^32^ ^]^ For the Cr‐doped TI device with a top gate (**Figure** **11**a), the Fermi level of the bottom surface is always located inside the surface‐state conduction band, whereas the Fermi level of the top surface can be modulated to different positions (Figure 11b), for example, near the bulk valence band edge, near the Dirac point, or inside the surface‐state conduction band. Consequently, the carrier density of the top surface is tunable by the top‐gate voltage, while the carrier density of the bottom surface remains unchanged. When the top‐gate voltage is tuned from −10 to +3 V, the carrier density of the top surface is close to minimum, as shown in Figure 11c. Meanwhile, owing to the opposite spin momentum‐locking direction of the bottom and top surface carriers, the net spin‐polarized surface current reaches maximum, thereby resulting in a maximum SOT (Figure 11d). The SOT can be tuned by a factor of four with the electrical field.

Thus far, high SOT efficiencies have been observed in various TIs, including Bi_2_Se_3_, Bi*_x_*Se_1−_ *_x_*, (Bi_0.5_Sb_0.5_)_2_Te_3_.^[^ ^30^, ^101^, ^102^, ^103^, ^104^, ^105^, ^109^, ^110^, ^114^, ^115^, ^116^, ^117^, ^118^, ^119^ ^]^ The largest SOT efficiency has been obtained in (Bi_0.5_Sb_0.5_)_2_Te_3_, which is in excess of 400 at low temperatures.^[^ ^114^ ^]^ However, the reported SOT efficiencies show large discrepancies. For example, very different SOT efficiencies have been reported in Bi_2_Se_3_/FM heterostructures, such as, Bi_2_Se_3_/NiFe with 3.5,^[^ ^30^ ^]^ Bi_2_Se_3_/CoFeB with 0.047,^[^ ^101^ ^]^ and Bi_2_Se_3_/CoFeB with 18.6.^[^ ^103^ ^]^ This discrepancy might result from different film qualities, magnon scattering mechanisms, etc., which need further investigation.

### 3.3 Hybrid Graphene‐Transition Metal Dichalcogenide and Graphene‐Topological Insulator Heterostructures

Although TMDs and TIs are promising spin‐source materials, their relatively low electrical conductivities remain a critical issue for achieving high SOT efficiency. The most studied 2D material, graphene, has recently been proposed to overcome this issue via hybrid engineering. Graphene possesses a much higher electron mobility than other 2D materials. In terms of spintronic application, pristine graphene also exhibits compelling features as a spin transport tunnel material owing to its weak SOC, that is, the merits of long spin lifetime and spin diffusion distance. However, the weak SOC inevitably leads to inefficient spin current generation. Fortunately, previous studies have suggested that this weak SOC can be enhanced by chemical functionalization, adatom decoration, and proximity effect with other materials.^[^ ^36^, ^37^, ^38^ ^]^ The former two approaches have a disadvantage of reducing the electronic quality of graphene. The proximity effect strategy has been proven as an efficient remedy, especially for enhancing the SOC of graphene via interfacing it with strong SOC materials.^[^ ^38^ ^]^ It is expected that the combination of graphene with TMDs or TI materials can induce a significant SOC in graphene while simultaneously maintaining its high electron mobility. The utilization of these hybrid heterostructures facilitates the improvement of the SOT efficiency.

**Figure 12:**
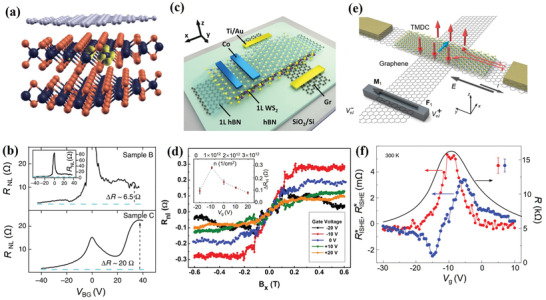
a) Schematic of a multilayer WS_2_/graphene heterostructure device. The highest unoccupied state of the sulfur vacancy is depicted in yellow, highlighting the W atoms closest to the vacancy. W, S, and C atoms are represented by dark‐grey, orange, and light‐grey spheres, respectively. b) Non‐local measurement signals for two different devices at 1.5 K and room temperature, respectively. a,b) Reproduced with permission.^[^ ^39^ ^]^ Copyright 2014, Springer Nature. c) Sketch of the vdW heterostructure of 1L WS_2_‐graphene. d) Gate dependence of the REE spin signals Hanle precession measured (at *T* = 4.2 K). The inset is the magnitude of the REE spin signal versus *V*_g_. c,d) Reproduced with permission.^[^ ^120^ ^]^ Copyright 2019, American Chemical Society. e) Illustration of the measurement concept. f) Spin‐to‐charge interconversion for the inverse SHE (red) and the spin‐galvanic effect (blue) as functions of *V*_g_. e,f) Reproduced with permission.^[^ ^121^ ^]^ Copyright 2020, Springer Nature.

Among the TMDs, WS_2_ is suggested to be a suitable substrate for graphene because of its comparable work function and strong SOC. Meanwhile, the presence of defects in WS_2_ could be helpful for the creation of electronic states that overlap in energy with the electronic states of graphene. Experimentally, when bringing graphene into proximity with WS_2_, the SOC strength has been demonstrated to be enhanced by three orders of magnitude (**Figure** **12**a,b).^[^ ^39^ ^]^ The SOC enhancement of graphene/WS_2_ is attributed to the proximity effect, which occurs when the sulfur vacancies induce gap states in the WS_2_. In addition to the proximity effect, the interface interaction also plays an important role in modifying the electronic properties of graphene. With a WS_2_ substrate, Wang et al. demonstrated a strongly enhanced SOC strength in graphene using weak antilocalization measurements.^[^ ^40^ ^]^ The induced SOC was attributed to the hybridization between graphene and substrate orbitals, which is a pure interface interaction. Additionally, proximity‐induced enhanced SOC also provides a possible route for tuning the charge‐spin interconversion in graphene. In monolayer WS_2_/graphene heterostructures, Ghiasi et al. first observed the charge‐to‐spin conversion based on REE (Figure 12c).^[^ ^120^ ^]^ The strong electric field dependence of REE spin signal shows the efficient tunability of spin generation (Figure 12d). Subsequently, a similar study was conducted in monolayer graphene/WS_2_ heterostructures by Benítez et al. (Figure 12e), who reported a strongly enhanced and tunable room‐temperature spin‐to‐charge conversion with spin precession experiments.^[^ ^121^ ^]^ Both conversion magnitude and sign can be tailored via electrostatic gating (Figure 12(f)). In addition to WS_2_/graphene, enhanced SOC and charge–spin interconversion have also been experimentally demonstrated in graphene/MoS_2_, graphene/MoTe_2_, graphene/TaS_2_ and graphene/WSe_2_ heterostructures.^[^ ^34^, ^41^, ^42^, ^122^ ^]^

**Figure 13:**
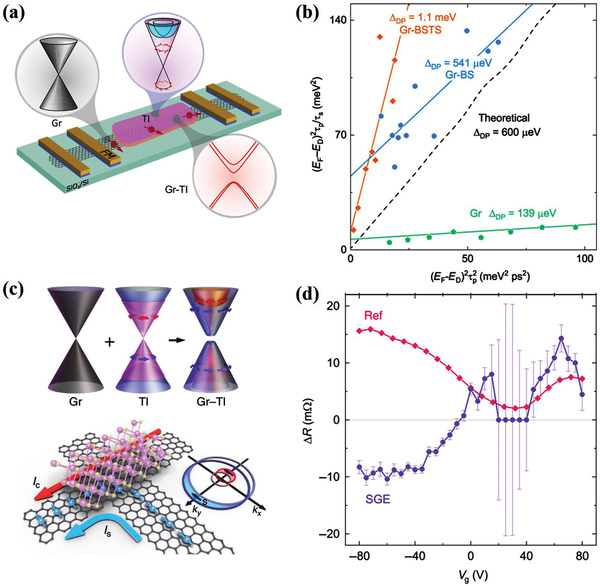
a) Schematic illustration of a graphene‐TI heterostructure. b) Estimation of SOC strength in graphene‐SiO_2_ (Gr, green), graphene‐Bi_2_Se_3_ (Gr‐BS, blue), and graphene‐Bi_1.5_Sb_0.5_Te_1.7_Se_1.3_ (Gr‐BSTS, orange) devices. The black dashed line shows the expected scaling based on ab initio simulations. a,b) Reproduced with permission.^[^ ^123^ ^]^ Copyright 2018, American Association for the Advancement of Science. c) Schematics of the band structures of pristine graphene (Gr), topological insulator (TI), and a Gr‐TI heterostructure. Owing to proximity‐induced spin‐orbit interaction, graphene develops Rashba spin‐split bands and acquires a spin texture that is different from the spin texture of the TI surface states. d) Amplitudes of the spin‐galvanic effect signal (purple) and scaled reference Hanle signal (pink) as functions of the gate voltage *V*_g_. c,d) Reproduced under the terms of the Creative Commons CC‐BY license.^[^ ^35^ ^]^ Copyright 2020, The Authors. Published by Springer Nature.

TIs and graphene have similar linear energy band dispersion near the Dirac point. In TI‐graphene hybrid heterostructure, a large Edelstein effect has been predicted.^[^ ^33^ ^]^ Such hybrid structures can significantly enhance spin accumulation and could be ideal spin sources for SOT devices. Experimentally, proximity‐induced SOC was recently observed in graphene/TI heterostructures in a spin‐polarized electron transport experiment (**Figure** **13**a).^[^ ^123^ ^]^ Two TI materials with different doping levels were used to construct two heterostructures, that is, Bi_2_Se_3_ and Bi_1.5_Sb_0.5_Te_1.7_Se_1.3_. Afterward, spin diffusion length and spin lifetime were extracted to evaluate the SOC strength. Compared with pristine graphene, spin diffusion length and spin lifetime were significantly reduced in the graphene/TI heterostructures. This suppression is attributed to strong proximity‐induced SOC in the graphene. Furthermore, in contrast to pristine graphene, the induced SOC strength was enhanced by nearly an order of magnitude (Figure 13b). Similar to graphene/TMDs heterostructures, proximity‐induced SOC is expected to be gate‐tunable in graphene/TI heterostructures. Khokhriakov et al. observed gate‐tunable SOC phenomenon in graphene/(Bi_0.15_Sb_0.85_)_2_Te_3_ heterostructure at room temperature.^[^ ^35^ ^]^ They studied the SOC by spin‐galvanic effect, which describes the conversion of spin current into charge current (Figure 13c). The clear spin‐galvanic effect signals confirm the existence of strong SOC, which is attributed to proximity‐induced Rashba spin texture in the hybrid graphene‐TI bands. This induced Rashba SOC makes both the amplitude and sign of the spin‐galvanic effect signal to be strongly tunable by the electric field, as shown in Figure 13d.

The enhancement of SOC in graphene/TMD and graphene/TI heterostructures provides a new building block for spin current generation. In conjunction with FM materials, current‐induced SOT in hybrid graphene‐based heterostructures can be modulated by an electrical field.^[^ ^124^ ^]^ These artificial structures may promote the implementation of electric field‐controllable SOT devices.

## 4 Van der Waals Ferromagnets and Antiferromagnets for Spin‐Orbit Torque

In addition to the non‐magnetic layer, ferromagnetic layer also plays a critical role in the realization of efficient SOT devices. First, its SOT strength is inversely proportional to its saturation magnetization and thickness. Second, for switching applications, such as SOT‐MRAM, the magnetization switching is related to the magnetic anisotropy of FM. Thus, one can further improve the SOT efficiency and performance of SOT devices by engineering the ferromagnetic layer. However, it remains challenging to prepare high‐quality ultra‐thin ferromagnets using traditional magnetic materials. Particularly, PMA magnet typically results from the interface effect. Furthermore, the high electrical conductivity of traditional ferromagnets also reduces the current efficiency of heterostructures.

The newly discovered vdW magnetic crystals provide exciting choices for SOT devices. VdW magnets are expected to inherit several merits of 2D materials, such as, flexibility and susceptibility to external stimuli. Most of them possess relatively low electrical conductivities; hence, they can increase the effective current in the NM layer. Furthermore, the flat surface and free‐of‐crystal mismatch are ideal for heterostructure construction. Till date, a number of vdW magnetic crystals have been experimentally confirmed, including ferromagnets such as, Cr_2_Ge_2_Te_6_, CrI_3_, Fe_3_GeTe_2_(FGT), and VSe_2_, and antiferromagnets (AFMs) such as, MPX_3_ (M = Fe, Mn, Ni; X = S) and CrCl_3_, MnBi_2_Te_4_.^[^ ^46^, ^47^, ^125^, ^126^, ^127^, ^128^, ^129^, ^130^ ^]^ Experimentally, ferromagnetic vdW crystals have been employed to construct conceptual spintronic devices. For instance, FGT and CrI_3_ have been successfully used to assemble FGT/hBN/FGT and graphene/CrI_3_/graphene magnetic tunnel junctions. Their reported TMR values are in excess of 10 000%.^[^ ^131^, ^132^, ^133^, ^134^, ^135^ ^]^ Most importantly, some ferromagnetic vdW crystals exhibit gate‐tunable magnetic properties in the 2D limit. Such unique magnetic properties could be very helpful for improving the performance of SOT devices. In the following section, we will summarize the recent progress of gate‐tunable magnetic properties and SOT research of vdW magnets

### 4.1 Gate‐Tunable Van der Waals Ferromagnets

**Figure 14:**
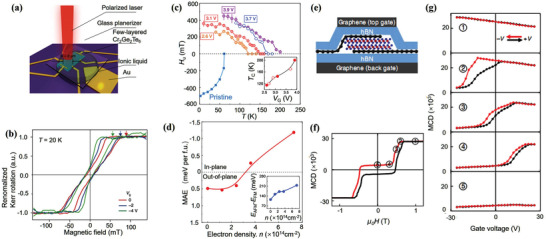
a) Schematic illustration of the experimental set‐up for the Kerr measurement of a micrometer‐sized Cr_2_Ge_2_Te_6_ device using ionic liquid. b) Renormalized Kerr angle measured at 20 K with fixed ionic gate voltages of 0, −2, and −4 V. The colored arrows indicate the saturation field for the loops measured at each *V*_g_. a,b) Reproduced with permission.^[^ ^137^ ^]^ Copyright 2018, Springer Nature. c) Uniaxial magnetic anisotropy field *H*_u_ as a function of gate bias for the Cr_2_Ge_2_Te_6_ device. The squares represent the values measured for a pristine bulk sample, whereas the filled and open circles represent the data from electric double‐layer transistor. Inset: Dependence of *T*_C_ on *V*_G_. d) Calculated magnetic anisotropy energy versus electron density. Inset: Change of total energy of ferromagnetic and antiferromagnetic spin states (*E*_AFM_ − *E*_FM_) at different doping densities. c,d) Reproduced with permission.^[^ ^138^ ^]^ Copyright 2020, Springer Nature. e) Schematic side view of a dual‐gate bilayer CrI_3_ field‐effect device. f) Magnetic circular dichroism (MCD) of bilayer CrI_3_ versus magnetic field under zero‐gate voltage at 4 K. g) Gate‐voltage control of MCD of bilayer CrI_3_ at 4 K. The black and red curves denote forward and backward sweep directions, respectively. e,f) Reproduced with permission.^[^ ^143^ ^]^ Copyright 2018, Springer Nature.

Among the discovered vdW ferromagnets, Cr_2_Ge_2_Te_6_, and CrI_3_ are ferromagnetic insulators that reduce the current shunt in SOT devices. Moreover, they both exhibit a PMA. Down to a few layers, their magnetic properties usually depend on the layer number.^[^ ^43^, ^136^ ^]^ For few‐layer Cr_2_Ge_2_Te_6_, the coercivity and saturation field are electrically tunable via ionic liquid gating (**Figure** **14**a,b).^[^ ^137^ ^]^ Very recently, Verzhbitskiy et al. showed that the transition temperature *T*_c_ and magnetic easy axis of thin Cr_2_Ge_2_Te_6_ can be modulated by electrostatic gating in an electric double‐layer transistor device.^[^ ^138^ ^]^ In addition to the significantly enhanced *T*_c_ (up to 200 K), the out‐of‐plane easy axis can be tuned to the in‐plane direction via electrostatic doping (Figure 14c,d). Although optical methods are usually used to characterize the magnetic properties of Cr_2_Ge_2_Te_6_ with a bandgap ≈0.7 eV,^[^ ^139^ ^]^ electrical transport measurement, which is important for studying SOT, has been explored to study the magnetic properties of Cr_2_Ge_2_Te_6_/Pt heterostructure.^[^ ^140^ ^]^ The Cr_2_Ge_2_Te_6_ induces ferromagnetism in Pt, resulting in anomalous Hall effect.

Compared to the small PMA in Cr_2_Ge_2_Te_6_ (10^5^ erg cm^−3^), CrI_3_ shows more robust PMA that is down to the monolayer limit. More interestingly, few‐layer CrI_3_ shows an intriguing magnetic phase associated with the number of layers.^[^ ^45^ ^]^ Monolayer and trilayer CrI_3_ were demonstrated to be ferromagnetically ordered by polar magneto‐optical Kerr effect microscopy. Meanwhile, bilayer CrI_3_ is an interlayer antiferromagnetic coupling with zero out‐of‐plane magnetization. However, the antiferromagnetic coupling is relatively weak and can be switched to the ferromagnetic order when the external field reaches a critical value. Magnetic properties in few‐layer CrI_3_ can also be electrically manipulated. Both electric field and electrostatic doping can effectively control its magnetic properties, such as, spin‐flipping magnetic field, coercive force, and transition temperature *T*_c_.^[^ ^141^, ^142^, ^143^ ^]^ In bilayer CrI_3_, the magnetic state can be conversed from the antiferromagnetic state to the ferromagnetic ground state by electrostatic doping without employing an external magnetic field (Figure 14e–g).^[^ ^143^ ^]^

**Figure 15:**
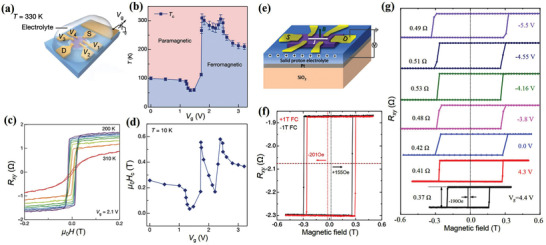
a) Schematic of the FGT device structure and measurement set‐up. b) Phase diagram of a trilayer FGT sample showing variations in the gate voltage and temperature. c) *R*_xy_ of a four‐layer FGT flake under a gate voltage of *V*_G_ = 2.1 V. d) Coercive field versus the gate voltage at *T* = 10 K. a–d) Reproduced with permission.^[^ ^51^ ^]^ Copyright 2018, Springer Nature. e) Schematic of the FGT Hall‐bar device on solid proton conductor. f) Exchange‐bias effect after 1‐T field cooling. g) Gate‐tuned ferromagnetism in FGT nanoflake (thickness: 115 nm). e–g) Reproduced with permission.^[^ ^150^ ^]^ Copyright 2020, American Physical Society.

Another popular gate‐tunable vdW ferromagnet is FGT, which possesses a reasonably high *T*_c_ and strong out‐of‐plane magnetocrystalline anisotropy.^[^ ^144^, ^145^, ^146^, ^147^ ^]^ The uniaxial magnetocrystalline anisotropy constant of FGT is on the order of ≈10^7^ erg cm^−3^, which is much larger than that of Cr_2_Ge_2_Te_6_. Such strong anisotropy allows the PMA to persist down to the monolayer level.^[^ ^44^, ^148^ ^]^ Unlike magnetic insulators Cr_2_Ge_2_Te_6_ and CrI_3_, FGT is metallic with a relatively large resistivity.^[^ ^44^, ^148^, ^149^ ^]^ The itinerant nature of ferromagnetism in FGT makes its magnetism more susceptible to conduction electrons that can, in turn, be tuned by the electrical field. A large range modulation of *T*_c_ has been experimentally demonstrated in few‐layer FGT devices with ionic gates (**Figure** **15**a).^[^ ^51^ ^]^ The *T*_c_ of few‐layer FGT is ≈100 K without the gate voltage. Meanwhile, a positive gate voltage can raise the *T*_c_ value above 300 K (Figure 15b,c). The coercive field also exhibits similar gate voltage dependence (Figure 15d). In addition to *T*_c_ and coercive force, the interlayer coupling in FGT has been recently shown to be tunable via the protonic gate (Figure 15e).^[^ ^150^ ^]^ Because of the coexistence of antiferromagnetic and ferromagnetic phases, the enhanced interlayer coupling induces a very large exchange bias field when a large positive voltage is applied (Figure 15f,g).

### 4.2 Van der Waals Ferromagnets for Spin‐Orbit Torque

**Figure 16:**
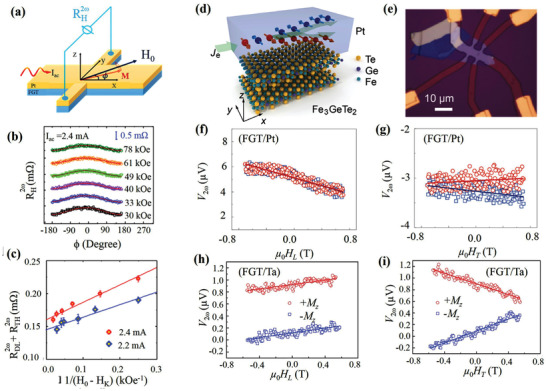
a) Schematic illustration of second harmonic Hall measurement in a FGT/Pt device. b) Second harmonic Hall resistance of a FGT (23 nm)/Pt(5 nm) device as a function of azimuthal angle *φ* for *I* = 2.4 mA. c) Effective SOT field *H*_DL_ for different currents. The red and blue solid lines represent the fit to the theoretical model. a–c) Reproduced with permission.^[^ ^151^ ^]^ Copyright 2019, American Chemical Society. d) Schematic view of the bilayer structure and optical image of the measured Hall bar device. Pt layer (top) is sputtered on top of the exfoliated FGT (bottom). f–i) Second harmonic voltages of FGT/Pt and FGT/Ta for the longitudinal effective field *H*_L_ (magnetic field along the x‐axis) and transverse effective field *H*_T_ (magnetic field along the y‐axis). d–i) Reproduced with permission.^[^ ^153^ ^]^ Copyright 2019, American Association for the Advancement of Science.

Atomically thin and gate‐tunable vdW ferromagnets provide unprecedented possibilities for the realization of high‐efficiency SOT devices. Particularly, FGT can potentially replace the conventional magnetic thin film owing to its higher *T*_c_ and stronger PMA. Indeed, high SOT efficiency has recently been observed in FGT/Pt heterostructures (**Figure** **16**a–c), in which 5‐nm Pt was sputtered onto 15–23‐nm exfoliated FGT flakes.^[^ ^151^ ^]^ The SOT strength and magnetization switching were measured using the second harmonic Hall method and anomalous Hall effect, respectively. Similar to conventional metal ferromagnets, the *τ*_FL_ was negligible, and the *τ*_DL_ dominated in the FGT/Pt. The estimated SOT efficiency competes well with the highest value of the conventional bilayer CoFeB/Pt.^[^ ^152^ ^]^ Although the detailed reasons of such high SOT efficiency are yet to be deeply understood, the authors believe that a factor plays an important role, that is, excellent interface resulting from the atomically flat FGT surface.

Furthermore, Wang et al. quantitatively characterized the SOT effective fields in few‐layer FGT/Pt and FGT/Ta heterostructures (Figure 16d,e).^[^ ^153^ ^]^ The FGT flakes were exfoliated down to few layers, and current‐induced SOT effective fields were determined from the second harmonic voltages (Figure 16f–i). Interestingly, the *τ*_DL_ effective field *H*_DL_ in the FGT/Ta sample was smaller than that in the FGT/Pt sample, and the *τ*_FL_ effective field *H*_FL_ for these two samples were comparable. Note that in conventional ferromagnetic metal bilayers, the *τ*_DL_ and *τ*_FL_ effective fields of Ta‐based heterostructures are typically larger than those in Pt‐based heterostructures. The authors speculate that this discrepancy resulted from the smaller interfacial spin transparency in the FGT/Ta sample. Furthermore, similar efficient SOT was also demonstrated in thin Cr_2_Ge_2_Te_6_/Ta heterostructure.^[^ ^154^, ^155^ ^]^ With the help of the in‐plane field, the critical current for the magnetization switch was much lower than that for conventional metallic ferromagnets. However, it should be noted that these SOTs were obtained at low temperatures.

### 4.3 Van der Waals Antiferromagnets for Spin‐Orbit Torque Application

**Figure 17:**
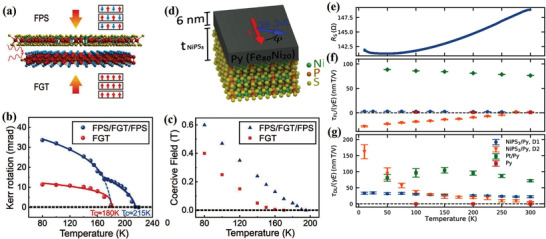
a) Magnetic ordering in vdW FGT and FPS thin flakes. b) Extracted Kerr rotation versus temperature for FGT (red curve) and FePS_3_/FGT/FePS_3_ (blue curve), with the solid lines showing fittings obtained using the function *α*(1−*T*/*T*_C_)^*β*^. c) Extracted coercive field versus temperature for FGT (red dots) and FePS3/FGT/FePS3 (blue dots). a–c) Reproduced with permission.^[^ ^163^ ^]^ Copyright 2020, Wiley‐VCH. d) Schematic of the bilayer NiPS_3_/Py used in the second‐harmonic Hall measurements. e) Sheet resistance *R*_S_ of the NiPS_3_/Py/Al(O*_x_*) Hall bar as a function of temperature. f) Field‐like torque *τ*_FL_ and g) damping‐like torque *τ*_DL_ as functions of temperature in the bilayer NiPS_3_/Py device, a Py reference sample, and a Pt/Py reference sample. d–g) Reproduced with permission.^[^ ^164^ ^]^ Copyright 2020, American Physical Society.

In contrast to ferromagnets, AFM, such as, MPX_3_ (M = Fe, Mn, Ni; X = S, Se) and CrCl_3_, are much more abundant.^[^ ^47^, ^127^, ^156^, ^157^, ^158^, ^159^ ^]^ Despite the well‐known difficulty of using AFMs because they do not have magnetization, AFMs have great potential in data storage and high‐frequency terahertz device applications. A typical application is to stabilize the magnetization of the “fixed layer” in magnetic tunnel junctions or spin valves. When assembling AFMs and ferromagnets in heterostructures, exchange bias is induced via exchange coupling.^[^ ^160^, ^161^, ^162^ ^]^ Such an exchange coupling can be tuned by the gate voltage in vdW AFM/FM heterostructures.^[^ ^161^ ^]^ This structure is useful in realizing efficient SOT devices, for instance, providing in‐plane exchange bias for SOT switching PMA magnets. Moreover, proximity‐coupling also plays an important role in these artificial structures. In a FGT/FePS_3_ heterostructure, the transition temperature and coercive field of FGT can be enhanced owing to the proximity of FePS_3_ (**Figure** **17**(a)–(c)).^[^ ^163^ ^]^ Furthermore, vdW AFMs can also serve as spin sources and provide a promising route for efficient SOT applications. A recent experimental work demonstrated that current‐induced SOT was present in a vdW AFM heterostructure via the interfacial effect (Figure 17d). As shown in Figure 17e–g, a large interfacial SOT was observed in the FePS_3_/Py heterostructure.^[^ ^164^ ^]^ The resulting spin conductivity of the *τ*_DL_ was ≈1 × 10^5^ (ℏ/2*e*)(Ωm)^−1^ at room temperature. The temperature dependence of SOT suggests that the magnetization order may have an inevitable influence on the SOT. Meanwhile, the detailed mechanism is not fully understood.

Finally, vdW magnetic materials have shown their great potential for SOT applications. Further work needs to be extended to all‐vdW material heterostructures. Combined with a variety of vdW spin current source, such as, gate‐tunable, strong SOC, and non‐trivial vdW materials, vdW magnetic materials can promote the implementation of energy‐efficient SOT devices.

## 5 Spin‐Orbit Torque‐Induced Magnetization Switching with Low Critical Current Density

It is of great importance to reduce the critical current density of SOT‐induced magnetization switching (hereafter, SOT switching) for application in SOT devices. In conventional HM/FM structures, the critical current density required for SOT switching is typically on the order of magnitude of 10^7^–10^8^ A cm^−2^. However, such high current densities are impractical for SOT applications. The utilization of vdW‐layered materials for the construction of NM/FM heterostructures is expected to reduce the critical current of SOT switching.

### 5.1 Spin‐Orbit Torque Switching in Transition Metal Dichalcogenide/Ferromagnetic

**Figure 18:**
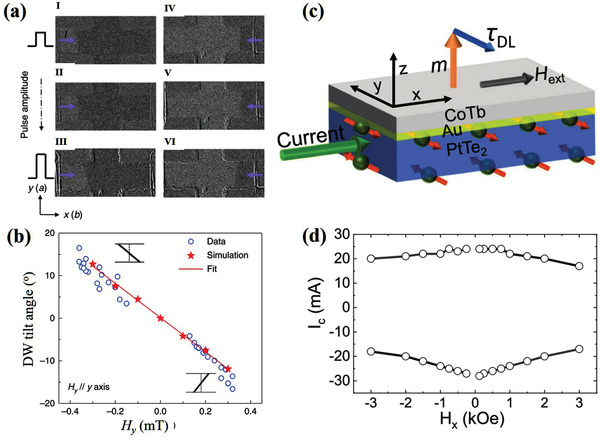
a) MOKE microscopy images for DW motion in WTe_2_/Py heterostructure and tilting with an increasing amplitude of current pulses. b) DW tilt angle as a function of easy‐axis external magnetic field *H*_y_ for both experimental and micromagnetic simulation results. The red line is a linear fit of the simulated data with *D* = −1.8 mJ m^−2^. a,b) Reproduced with permission.^[^ ^29^ ^]^ Copyright 2019, Springer Nature. c) Schematic layout of PtTe_2_/Au/CoTb stack and the SOT generated by the majority of current flowing in PtTe_2_. d) Switching phase diagram of the PtTe_2_/Au/CoTb heterostructure, where *I*_c_ is the critical switching current. c,d) Reproduced with permission.^[^ ^77^ ^]^ Copyright 2020, Wiley‐VCH.

All‐electric magnetization switching was recently demonstrated in WTe_2_/FM heterostructures, in which the magnetic layer was Py with in‐plane magnetization.^[^ ^29^ ^]^ The easy axis of Py was set along the a‐axis (or b‐axis), whereas the hard axis and current were set along the b‐axis (or a‐axis) to allow SOT switching without external magnetic field. When the current was along the a‐axis, the critical current density required for SOT switching was clearly lower than that in the b‐axis. These results indicate that *τ*_B_ increases the switching efficiency. Noticeably, current‐induced magnetization switching was completed via domain wall nucleation and propagation (**Figure** **18**a). Moreover, the critical current density was lower than 3 × 10^5^ A cm^−2^. Interestingly, experimental observation and micromagnetic simulation also suggested the existence of Dzyaloshinskii–Moriya interaction with a constant *D* = −1.78 ± 0.06 mJ m^−2^ (Figure 18b), which was comparable with that of Pt/Co. Subsequently, a similar field‐free SOT switching experiment was conducted using a MoTe_2_/Py heterostructure by the same group.^[^ ^77^ ^]^ The threshold current required for SOT switching was approximately 6.7 × 10^5^ A cm^−2^ in a rectangular MoTe_2_ (66.1 nm)/Py (6 nm) sample. This critical current density can be decreased by a factor of three through shape engineering, which is due to the decrease of the domain nucleation energy by a reasonable shape design. High‐efficiency SOT switching was also demonstrated in PtTe_2_/CoTb heterostructure (Figure 18c).^[^ ^86^ ^]^ The lowest critical current density was 3.1 × 10^6^ Acm^−2^ when in‐plane bias field was employed (Figure 18d). Recently, SOT switching was further extended to the WTe_2_/FGT heterostructure. By utilizing the strong SOC of WTe_2_, field‐free and efficient SOT switching were achieved at low temperature.^[^ ^165^, ^166^ ^]^

### 5.2 Spin‐Orbit Torque Switching in Topological Insulator/Ferromagnetic

**Figure 19:**
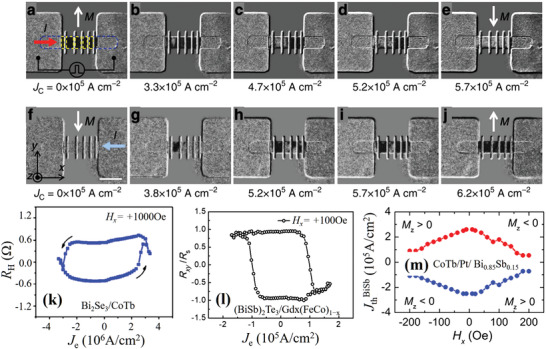
a–j) MOKE images of SOT‐driven magnetization switching in Bi_2_Se_3_/Py at zero magnetic field and room temperature. a–j) Reproduced under the terms of the Creative Commons CC‐BY license.^[^ ^91^ ^]^ Copyright 2017, The Authors. Published by Springer Nature. k) Room‐temperature SOT switching in Bi_2_Se_3_/CoTb. *J*_e_ is the average current density inside the Bi_2_Se_3_ layer. Reproduced with permission.^[^ ^105^ ^]^ Copyright 2017, American Physical Society. l) SOT switching with an in‐plane magnetic field *H_x_* of +100 Oe in the (BiSb)_2_Te_3_/Gd*_x_*(FeCo)_1−_ *_x_* system. Reproduced with permission.^[^ ^167^ ^]^ Copyright 2019, Wiley‐VCH. m) SOT magnetization switching by sweeping a DC current. Threshold switching current density *J* ^BiSb^_th_ as a function of in‐plane magnetic field *H_x_* for CoTb/Pt/Bi_0.85_Sb_0.15_. Reproduced under the terms of the Creative Commons CC‐BY license.^[^ ^168^ ^]^ Copyright 2020, The Authors. Published by Springer Nature.

Owing to the TSS‐induced large SOT efficiency of TI, the critical current density of SOT switching in TI/FM heterostructures can be further reduced. Ultra‐low current density required for SOT switching has been observed in TI/magnetic‐TI structures.^[^ ^114^ ^]^ By employing an in‐plane magnetic field, the critical current density was below 8.9 × 10^4^ A cm^−2^, which is much lower than that in bilayer HM/FM heterostructures. Moreover, electric field manipulation of the magnetization switching was also realized in magnetic TI.^[^ ^32^ ^]^ However, it must be noted that the low current density switching was obtained only at ultra‐low temperatures because of the low transition temperature of the magnetic TIs.^[^ ^32^, ^114^, ^117^ ^]^ Wang et al. found large SOT switching in TI/FM heterostructures at room temperature, in which a conventional ferromagnet alloy Py was used as the thin magnetic layer. The magnetic easy axis was set to be collinear with the spin polarization so that SOT switching could be achieved without employing an external magnetic field. In contrast to HM/FM, the critical current density was significantly reduced to 6 × 10^5^ A cm^−2^ (**Figure** **19**a–j).^[^ ^102^ ^]^

In terms of application of techniques, PMA magnets are preferred because of their better thermal stability and reduced critical current density. However, the main challenge is the deposition of FM with PMA onto the TIs. The PMA of commonly used magnetic materials strongly relies on the interfacial condition. When neighboring with TI, the PMA magnetic layer usually becomes a magnet with in‐plane magnetization anisotropy. Magnets with bulk PMA provide a feasible choice for the magnetic layer, such as transition metal–rare earth ferrimagnetic alloys Co*_x_*Tb_1−_ *_x_*, Gd*_x_*(FeCo)_1−_ *_x_*. Their magnetic properties can be modulated within a wide range of values via chemical composition engineering. Combining high transition‐temperature bulk PMA magnets with TI, efficient SOT switching has been achieved in Bi_2_Se_3_/CoTb heterostructures at room temperature.^[^ ^105^ ^]^ The critical current density for switching was approximately 2.8 × 10^6^ Acm^−2^ with a 1000 Oe in‐plane bias field (Figure 19k). Subsequently, much lower current density of SOT switching was realized at room temperature. The corresponding current density can be reduced to 1.2 × 10^5^ A cm^−2^ in a nearly compensated ferrimagnet Gd*_x_*(FeCo)_1−_ *_x_* (Figure 19m).^[^ ^167^ ^]^ In non‐epitaxial Bi_2_Sb_3_/CoTb, the critical current density is as low as 7 × 10^4^ A cm^−2^ (Figure 19m).^[^ ^168^ ^]^

**Figure 20:**
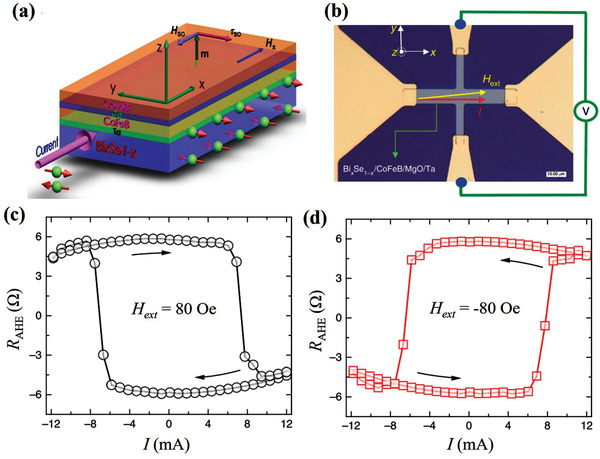
a) 3D schematic illustration of SOT in a Bi*_x_*Se_(1−_ *_x_*_)_/CoFeB heterostructure. *H*_ext_ is the in‐plane externally applied magnetic field. The red arrows represent the direction of the spin‐magnetic moment. b) Optical micrograph of the fabricated Hall cross bar with schematic illustrations of the Hall measurement set‐up. c,d) SOT‐induced switching of the magnetization in the presence of constant in‐plane bias fields. a–d) Reproduced with permission.^[^ ^103^ ^]^ Copyright 2018, Springer Nature.

Recently, interfacial PMA magnetic layer was achieved by a thin insertion layer. Mahendra et al. successfully prepared a PMA CoFeB layer by sputtering and demonstrated SOT switching in Bi*_x_*Se_(1‐_ *_x_*_)_/CoFeB heterostructure (**Figure** **20**a,b).^[^ ^103^ ^]^ The interfacial PMA magnetic layer CoFeB was obtained via the Ta insertion layer between the CoFeB and TI layers. With an 80 Oe assistive in‐plane bias magnetic field, magnetization switching was achieved at 7.2 mA (≈4.3 × 10^5^ A cm^−2^) (Figure 20c,d). The required current density was approximately two orders of magnitude smaller than that of the Ta sample. Despite the positive role of PMA formation by the Ta insertion layer, a drawback is the significantly increased spin density dissipation owing to spin‐flip scattering. In contrast to heavy metal Ta, light metal Mo possesses smaller SOT efficiency and longer spin diffusion length. By replacing the Ta insertion layer with a Mo spacer, interfacial PMA was also achieved without significant influence of the spin current generated from the TI. Owing to the large SOT efficiency, the critical current density was as low as ≈3 × 10^5^ A cm^−2^ in the (BiSb)_2_Te_3_/Mo/CoFeB structure. Similarly, interfacial PMA magnetic layer and efficient SOT switching have been obtained in Bi*_x_*Te_1−_ *_x_* /Pt/Co/Pt, Bi_2_Se_3_/Co/Pt.^[^ ^169^, ^170^ ^]^

**Figure 21:**
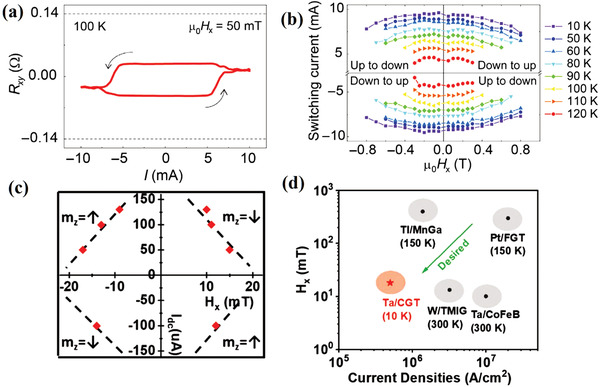
a) Current‐driven perpendicular magnetization switching in a bilayer FGT/Pt device for in‐plane magnetic fields of 50 mT at 100 K. b) Switching‐phase diagram with respect to the in‐plane magnetic fields and critical switching currents at different temperatures. In the bilayer device, applying a current of 1 mA corresponds to a current density of 1.85 × 10^10^ A m^−2^ in the Pt layer. a,b) Reproduced with permission.^[^ ^153^ ^]^ Copyright 2019, American Association for the Advancement of Science. c) Phase diagram of m_z_ in a Cr_2_Ge_2_Te_6_/Ta device for applied combinations of *I*_dc_ and *H_x_* at 4 K. d) Current densities and in‐plane fields required for SOT switching of previously reported magnetic heterostructures in comparison to those of Cr_2_Ge_2_Te_6_/Ta. Reproduced with permission.^[^ ^154^ ^]^ Copyright 2020 Wiley‐VCH.

Compared to conventional ferromagnetic materials, vdW ferromagnets have atomically flat interfaces and ultra‐thin thicknesses, as well as gate‐tunable magnetic properties. These features are suggested to be helpful for high‐efficiency SOT switching. Few studies have revealed the low critical current density required for SOT switching in vdW ferromagnets/HM heterostructures, such as, FGT/Pt with 8.3 × 10^6^ A cm^−2^ and Cr_2_Ge_2_Te_6_/Ta with 5 × 10^5^ A cm^−2^, as shown in **Figure** **21**a–d.^[^ ^153^, ^154^ ^]^ Although these SOT switchings are obtained at relatively low temperatures, they show great prospect in efficient SOT switching. Finally, we summarize the critical current density required for SOT switching in various vdW heterostructures in **Table** **1**.

**Table 1:** SOT switching critical current density of various vdW heterostructures

Material	Magnetic system	T [K]	*J*_c_ [A cm^−2^]	References
WTe_2_/Py	IMA	RT	≈3.0 × 10^5^	[29]
MoTe_2_/Py	IMA	RT	≈6.7 × 10^5^	[77]
PtTe_2_/CoTb	PMA	RT	≈3.1 × 10^6^	[86]
(BiSb)_2_Te_3_/Cr‐(BiSb)_2_Te_3_	PMA	1.9	≈8.9 × 10^4^	[114]
Bi_2_Se_3_/Py	IMA	RT	≈6.0 × 10^5^	[102]
Bi_2_Se_3_/CoTb	PMA	RT	≈2.8 × 10^6^	[105]
Bi_2_Se_3_/Gd*_x_*(FeCo)_1−_ *_x_*	PMA	RT	≈1.2 × 10^5^	[167]
Bi_2_Sb_3_/CoTb	PMA	RT	≈7.0 × 10^4^	[168]
Bi*_x_*Se_(1‐_ *_x_*_)_/CoFeB	PMA	RT	≈4.3 × 10^5^	[103]
(BiSb)_2_Te_3_/Mo/CoFeB	PMA	RT	≈3.0 × 10^5^	[104]
Bi*_x_*Te_1−_ *_x_*/Pt/Co/Pt	PMA	RT	≈6.5 × 10^5^	[169]
Bi_2_Se_3_/Co/ Pt	PMA	RT	≈2.5 × 10^6^	[170]
FGT/Pt	PMA	180	≈2.5 × 10^7^	[151]
FGT/Pt	PMA	120	≈8.3 × 10^6^	[153]
Cr_2_Ge_2_Te_6_/Ta	PMA	10	≈5.0 × 10^5^	[154]

RT, room temperature; IMA, in‐plane magnetic anisotropy; PMA, perpendicular magnetic anisotropy; *J*_c_, critical current density for SOT switching.

## 6 Conclusion and Outlook

Current‐induced SOT offers a powerful tool for efficiently controlling magnetization without external magnetic fields, and it allows the realization of high‐performance spintronic devices, such as SOT‐MRAM. To realize power‐efficient SOT devices, considerable efforts have been made to enhance the SOT efficiency and reduce the critical current density required for SOT switching. A number of vdW‐layered materials have been explored for this purpose, including TMDs, TIs, graphene‐based heterostructures, and vdW magnets. TMDs and TIs provide an attractive avenue for the realization of large SOT efficiency. First, they show very efficient charge‐to‐spin conversion. Second, the SOT in these materials can be tuned by designing the crystal symmetry and electrical field. Thus, unconventional SOT is allowed in TMDs with reduced crystal symmetry. This novel SOT makes the magnetization switching more efficient. Finally, TMDs or TIs can significantly enhance the SOC of graphene via the proximity effect. These hybrid heterostructures not only maintain the merit of high electron mobility and strong SOC but also show tunability by electrical field. Thus, they are promising spin current‐source materials.

The newly discovered vdW magnets provide further opportunities for SOT devices. On the one hand, from metal to semiconductor, diverse ultra‐thin and gate‐tunable vdW ferromagnets have been demonstrated to reduce power consumption and improve the performance of SOT devices. On the other hand, vdW AFMs have been shown to be efficient spin sources owing to the interface effect. Finally, we discussed the SOT switching critical currents in various vdW heterostructures. TMDs and TIs exhibited much lower critical currents than the typical value of HM. Hence, these vdW materials have unprecedented potential implementation in efficient spintronic devices.

Continuous endeavors need to be devoted to the field of SOT in vdW materials. First, as spin current‐source materials, TMDs and TIs exhibit considerable SOT efficiencies. However, their microscopic origin is not explicitly clear at the moment. Additionally, novel SOTs have been observed in some TMDs, such as out‐of‐plane antidamping‐like SOT and in‐plane field‐like SOT, which cannot be explained by the conventional SOC effect, such as, SHE and REE. The detailed mechanisms need further experimental and theoretical studies. Furthermore, most reported TMDs and TIs have small electrical conductivities, and it is challenging to find TMDs and TIs with high conductivities and strong SOCs.

Although graphene‐TMD (or TI) hybrid structures showed enhanced SOC, the experimental investigation of current‐induced SOT in TMDs/graphene/FM or multiple‐layer TIs/graphene/FM heterostructures, is still lacking. Furthermore, the role of the interface in vdW NM/FM heterostructures remains unclear. For example, when inserting graphene between the TMD (TI) and FM layer, it is not clear whether the interface transparency will strongly affect the SOT efficiency.

For the choice of magnetic layer, atomically thin vdW‐layered magnetic crystals are promising candidates. Unfortunately, at the current stage, most of them have relatively low Curie temperatures *T*_C_. Continuous efforts should be made to explore high *T*_C_ vdW magnets. For example, the temperature can be increased by optimizing the exchange interaction and the magnetic anisotropy. Assembling the vdW heterostructure with other materials may also be helpful in enhancing the *T*_C_ of vdW magnets.^[^ ^163^, ^171^, ^172^ ^]^ In addition, doping, such as, Ga implantion and Co doping were recently demonstrated to be effective to increase the *T*_C_.^[^ ^173^, ^174^, ^175^ ^]^

Finally, wafer‐scale scalability of vdW materials is still an important issue. At present, vdW materials are usually prepared by mechanical exfoliation in SOT devices, which is difficult to integrate with the modern semiconductor technology. Although the latest progress in the manufacture of large‐area vdW materials advances the spintronic development for industry applications,^[^ ^44^, ^86^ ^]^ long‐term technically fundamental challenges still need to be addressed, such as, wafer‐scale growth, efficient transfer, and high‐quality heterostructrue assembling.

In conclusion, despite several challenges to be addressed, new opportunities in vdW material‐based SOT devices attract increasing interests in both fundamental research and technology applications. The progresses achieved in vdW materials, such as, large SOT efficiency, controllable SOC, and gate‐tunable magnetism, can promote the development of next‐generation efficient spintronic devices. We expect vdW materials to pave the way for more ambitious development of SOT devices.

## Conflict of Interest

The authors declare no conflict of interest.
